# Supplementary material for: The Systems Biology Research Tool: evolvable open-source software
Source: BMC Syst Biol. 2008 Jun 29;2:55. doi: 10.1186/1752-0509-2-55 (PMC2446383; doi:10.1186/1752-0509-2-55)
Supplement: Additional file 1 — SBRT Archive. An archive of the current version of the Systems Biology Research Tool. [file 1752-0509-2-55-S1.zip › sbrt-1.4.0/doc/developers_guide/api/sbrt/ext/R/stat/class-use/RCorrelator.Result.html]

Uses of Class sbrt.ext.R.stat.RCorrelator.Result


|  |  |  |
| --- | --- | --- |
| **Overview** \| **Package** \| **Class** \| **Use** \| **Tree** \| **Deprecated** \| **Index** \| **Help** | |  |
| PREV   NEXT | **FRAMES**    **NO FRAMES**     **All Classes** |  |


---


## **Uses of Class sbrt.ext.R.stat.RCorrelator.Result**

| Packages that use RCorrelator.Result | |
| --- | --- |
| **sbrt.ext.R.stat** | Provides classes that allow communication with R for the purposes of statistics. |
| **sbrt.shell.mng.stat** | Provides classes and interfaces for creating statistical process managers. |

Uses of RCorrelator.Result in sbrt.ext.R.stat

| Methods in sbrt.ext.R.stat that return RCorrelator.Result | |
| --- | --- |
| `RCorrelator.Result` | `RCorrelator.corr(java.util.Collection<java.lang.Double> x, java.util.Collection<java.lang.Double> y)`             Computes and returns the correlation results for the provided values. |
| `RCorrelator.Result` | `RCorrelator.corr(java.lang.Double[] x, java.lang.Double[] y)`             Computes and returns the correlation results for the provided values. |
| `RCorrelator.Result` | `RCorrelator.corr(java.util.Map<? extends V,java.lang.Double> xValues, java.util.Map<? extends V,java.lang.Double> yValues)`             Computes and returns the correlation statistic of the provided x and y values. |

Uses of RCorrelator.Result in sbrt.shell.mng.stat

| Methods in sbrt.shell.mng.stat that return types with arguments of type RCorrelator.Result | |
| --- | --- |
| `CorrelationAnalysis<java.lang.String,RCorrelator.Result>` | `CorrelationManager.getProcess()`             Returns the process controlled by this process manager. |

---


|  |  |  |
| --- | --- | --- |
| **Overview** \| **Package** \| **Class** \| **Use** \| **Tree** \| **Deprecated** \| **Index** \| **Help** | |  |
| PREV   NEXT | **FRAMES**    **NO FRAMES**     **All Classes** |  |


---
